# Local anesthesia for cochlear implant surgery: a possible alternative

**DOI:** 10.1590/S1808-86942010000500005

**Published:** 2015-10-22

**Authors:** Rogério Hamerschmidt, Marcos Mocellin, Alexandre Camilloti Gasperin, João Luiz Garcia de Faria, Guilherme Trevizan, Gislaine Richter Minhoto Wiemes, Valéria Kutianski

**Affiliations:** 1 MSc, Professor of Otorhinolaryngology - University Hospital of the Federal University of Paraná; 2 PhD, Full professor of Otorhinolaryngology - University Hospital of the Federal University of Paraná; 3 MSc; Physician of the Otology Department - Instituto Paranaense de Otorrinolaringologia; 4 PhD, ENT at the Otorhinolaryngology Department - University Hospital - Federal University of Paraná; 5 Expert. Physician - Department of Otology - Instituto Paranaense de Otorrinolaringologia; 6 MSc - Speech and Hearing Therapist - Department of Otorhinolaryngology - University Hospital - Federal University of Paraná; 7 Expert - Speech and Hearing Therapist - Otology Department - Instituto Paranaense de Otorrinolaringologia. Instituto Paranaense de Otorrinolaringologia

**Keywords:** anesthesia, cochlear implantation, conscious sedation.

## Abstract

**Abstract:**

The aim of this paper is to illustrate the possibility of performing a cochlear implant surgery with local anesthesia and sedation, the anesthetic technique and the advantages of that in comparison to a general anesthesia.

**Aims:**

prospective study demonstrating the possibility of doing cochlear implant surgery under local anesthesia and sedation.

**Materials and methods:**

we describe three successful cases operated under local anesthesia, including neural telemetry and the conditions the patient presented after the surgery, with a very good recovery and no complications during and after the procedure.

**Results:**

these three surgeries show the possibility of doing the cochlear implant surgery with this kind of anesthesia, with a fast recovery, no symptoms of dizziness and vomiting after the surgery, and very few complaints from the patient.

**Conclusion:**

local anesthesia with sedation for cochlear implant surgery in adults is a very good alternative for lowering the morbidity for the patient. It bears fewer risks, low costs for the hospital, with a very good procedure control, being very useful for older patients or the ones that have contraindications for general anesthesia. Clinical trial register - IC - 026 (clinicaltrials.gov)

## INTRODUCTION

Cochlear implant surgery is enjoying a rapid growth, thanks to improvements in implant quality, less invasive procedures and greater communication about this type of treatment for hearing loss. Cochlear implants are extremely expensive prosthesis, which can partially replace cochlear function.^1^ The procedure today is much faster and less invasive than it was some years ago, with fewer incisions and lower patient morbidity. Nonetheless, some problems still occur, especially in elderly patients, concerning anesthesia^1^. Frequently, elderly patients have comorbidities such as arterial hypertension and diabetes mellitus, which can increase bleeding during surgery under general anesthesia, besides a greater likelihood of cardiac arrhythmias and tracheal intubation difficulties - in heavier patients with short neck. Moreover, general anesthesia brings about more costs to the hospital, patient recovery in the immediate post-op is more symptomatic, and the risks are higher. Nausea and vomiting are very common under this type of anesthesia, especially in the first hours, increasing the need for anti-vomiting medication and hospital stay. In younger adult patients, who could undergo general anesthesia, the major advantage of local anesthesia is the postoperative, with less nausea and vomiting and early discharge; therefore, with a lower risk of hospital infection, besides a greater acceptance by the patient concerning this type of anesthesia.

This paper stresses the possibility of doing the cochlear implant surgery under local anesthesia and sedation, the drugs used and the advantages of this technique in adult patients.

## MATERIALS AND METHODS

This paper was carried out based on three patients submitted to cochlear implant surgery under local anesthesia and sedation. It was approved by the Ethics in Research Committee of our Institution on July 10, 2009, protocol 003/2009.

All the patients had profound, irreversible, bilateral hearing loss, which did not respond to conventional hearing devices and were selected by the institution's cochlear implant team, following national criteria from the Ministry of Health in order to indicate the surgery. Each one of them was submitted to all necessary tests, including tonal audiometry, immittance measures, brainstem auditory evoked potential, vector-electronystagmography, left ear and mastoid CT-Scan and inner ear MRI with cochlear three-dimensional reconstruction. Moreover, we did all the pre-op workup, including CBC, coagulogram and electrocardiography, depending on the associated comorbidities, and evaluation with the anesthesiologist when the issues pertaining to this type of anesthesia were discussed, and what the patient could feel during the procedure. The anesthesiologist approaches the issues associated with the sedation, the fact that the patient will feel the physician manipulating the ear at the time of the procedure, explaining that this is normal and that there is no problem, which is different from general anesthesia, in which the patient does not see anything and does not feel anything. It is also explained that at the time of the neural telemetry, the patient can feel some stimulus, like the sound of an alarm or a mild discomfort, absolutely normal, and which also helps show that the electrode is correctly positioned, providing a hearing sensation to the patient.

The first patient was male, 35 years of age, with bilateral congenital profound hearing loss, implanted on the right ear with a Sonata, Med-EL device.

The second patient was a 28 year-old woman, with mild cochlear ossification, idiopathic, implanted on the left ear with a Nucleus Freedom from Cochlear Corporation, with total implant insertion, despite ossification.

The third patient, also a 22 year-old female with congenital hearing loss, implanted on the right ear with a Nucleus Freedom device, without complications.

The patients, all adults, were chosen for their stable emotional status, with all the necessary explanation about the anesthesia and the sedation, promptly accepted by them. The choice was based on emotional issues, patient preference for this type of anesthesia and the lack of contraindications, such as the medication used, important emotional status or a negative emotional status concerning this type of anesthesia.

The surgical technique used was the traditional one, with a retroauricular access, with a 3cm incision, simple mastoidectomy, posterior tympanotomy, cochleotomy and implant insertion, creating a niche for the internal unit. The technique was not changed on account if this being a local anesthesia.

The anesthetic protocol involved complete evaluation of the patient's general health status, investigation of other pathologies and an explanation by the anesthesiologist at the time of the procedure regarding what was about to happen - using jests and lip reading. The patient did not receive pre-anesthetic medication. Monitoring included electrocardiogram and pulse oximetry. All the patients received 1ucg/kg fentanyl, 0.5 mg/kg meperidine, 5 mg midazolam and 2 ucg/kg clonidine at the time of the anesthesia induction. Nasal oxygen was given at the rate of 3l/min.

After sedation, local anesthesia was done with xylocaine and adrenalin 1:50,000, in the retroauricular region, in the region of the niche created for the internal unit and in the four quadrants of the external auditory meatus. The pressure was kept normal during surgery, without inducing hypotension.

Neural telemetry was carried out without problems and the mild reaction the patients had: one of themblinked, the other moved the hands, a third did not show any reaction, despite telemetry being absolutely normal and in all cases showed proper cochlear nerve stimulus.

During surgery, the opioid dose is repeated if necessary, when the patient complains of some pain or discomfort, or if the patients started to wake up.

Other drugs routinely used are ondansetron 4 mg, repeated if needed; metoclopramide 10mg, defazolin 1g, dexamethasone 1mg/kg, dipirone 1g and ketorolac 30 mg.

The anesthetic reversion is done with naloxone 0.2 mg.

At the end of the surgery, the conventional dressing is done and the patient remains in the recovery room for about half-an-hour, afterwards being sent to the apartment, and then, three hours later the patient can go home. We prescribe paracetamol 750 mg, twice a day, meclizine 25 mg if needed; and antibiotic coverage with amoxicillin with clavulanic acid for 7 days. The remaining of the healing period and implant activation happen as usual.

## RESULTS

The surgeries lasted one hour and a half in average, and the patient should not make any moves which could damage the device. The surgical technique was exactly the same used under general anesthesia, in such a way that the surgeon is able to technically perform the procedure in a fast and safe way, and also the anesthesiology team is used to this type of surgery.

The three patients had their cochlear implant surgery without complications, not even in the critical moments such as the cochleostomy and neural telemetry, not showing any sign of restlessness at these times, and at the end of the procedure, after sedation reversion done by the anesthesiologist, they did not have nausea or vomits, having an eventful postoperative and being discharged after some hours. In the cases of general anesthesia, the patients takes longer to be fully awake and conscious, nausea and vomiting symptoms are stronger and hospital discharge takes some extra hours. The dressing was removed after two days, and the rest of the postoperative was conventional, and there were no differences as far as healing, hearing and implant activation goes, when compared to general anesthesia. Therefore, the greatest advantage is really during surgery, with lesser risk and immediate and late postoperative were regular.

## DISCUSSION

We started our cochlear implant service three years ago, finding many difficulties to show the skill and competence to the patients, procedure approval by the health insurance companies, and acquisition of all the necessary material in order to do the surgery, besides setting up a highly qualified team for the project. Today, we have a complete team with all the necessary professionals, working practically exclusively with cochlear implants.

In order to reach the best possible results, the entire team must work with perfection, and this includes a highly processional and skilled team of anesthesiologists. The anesthesia is a fundamental issue associated with the surgery, because both during it and afterwards, patient recovery depends on the immediate post-op symptoms and on the prompt patient recovery to return to his/her regular activities.

We decided to start doing cochlear implant surgery in adults under local anesthesia and sedation for many reasons. General anesthesia has a very high cost, making the health insurance plans to agree with the local anesthesia, but the main factor is patient safety. Under local anesthesia and sedation there is lower morbidity and less vomiting and nausea, and the patient goes home on the same day. Moreover, many patients feel safer for undergoing this procedure with this type of anesthesia, since general anesthesia still is a great fear faced by most of the patients. There is also the issue that our patients are becoming increasingly older, and many of them could not undergo the procedure under general anesthesia; with this new approach they could be eligible for cochlear implant surgery.

The anesthesiology service must also be fully prepared to deal with the issues associated with hearing loss, know how to approach the hearing impaired patient and also explain in a clear and objective way what will happen during the surgery, especially some discomfort the patient may feel during intraoperative neural telemetry.

We do not have many world literature papers regarding this topic since this is a worldwide innovative technique which started in our service. General anesthesia is a routine practice in most departments in the world for all otologic surgeries and also for other ENT subspecialties, and it is very likely because of this routine and habit that local anesthesia has never been considered. Of course, it all depends on the surgeon and also of the anesthesiologist, and since we are used to doing all chronic ear surgeries under local anesthesia and sedation, we decided to do the cochlear implant the same way, and this was greatly accepted by the patients.

The efficacy of the technique has been well established for other ear surgeries, such as mastoidectomies, stapedectomies and tympanoplasties. We have also performed some inner ear surgeries under this type of anesthesia, such as endolymphatic sac decompression. Nonetheless, for the cochlear implant surgery, there are many psychological and emotional aspects involved^2^. All these aspects must be well worked upon by the medical team, by speech and hearing therapists and especially by the psychologists, to help patients feel safer and this also influences the decision to undergo the surgery under local anesthesia and sedation, so that the surgery can be uneventful. Another issue is the electrical stimulus which telemetry is responsible for^3^. We had some fear that at this time the patient could have shown a stronger reaction, or move if it were not tolerated; however, at this time, the anesthesiologist increases the sedation even further, and telemetry happens without problems.^4^,^5^,^6^

## CONCLUSION

We have concluded that with this new possibility of doing cochlear implant surgery under local anesthesia is perfectly doable, with many advantages when compared to general anesthesia, and it opens a large possibility of surgeries for patients who could not be operated because of anesthetic problems.
